# Perceived harm of menthol cigarettes and quitting behaviors among menthol smokers in Minnesota

**DOI:** 10.1016/j.pmedr.2020.101269

**Published:** 2020-11-28

**Authors:** Paula A. Keller, Joanne D'Silva, Rebecca K. Lien, Raymond G. Boyle, John Kingsbury, Erin O'Gara

**Affiliations:** a ClearWay Minnesota^SM^, 8011 34th Ave S, Suite 400, Minneapolis, MN 55425, USA; b 655 19th Ave NE, Suite 100, Minneapolis, MN 55418, USA; c 2331 Roosevelt St, Minneapolis, MN 55418, USA; d Minnesota Department of Health, P.O. Box 64975, Minneapolis, MN 55164-0975, USA

**Keywords:** Smoking, Menthol, Risk, Harm perception, Quitting

## Abstract

Although overall smoking prevalence in Minnesota has declined, the proportion of current smokers who smoke menthol cigarettes has increased. While studies have examined associations between smokers’ perceived risks of smoking and quitting, similar studies on menthol smoking are lacking. This study examined whether perceived harm of menthol cigarettes was associated with menthol smokers’ quitting behaviors.

Data from the 2018 Minnesota Adult Tobacco Survey were examined. Respondents were categorized as current menthol smokers (n = 200), current nonmenthol smokers (n = 527), or nonsmokers (n = 5324). All were asked four questions to assess their perceptions of menthol cigarettes’ harm compared to nonmenthols. Sum scores were calculated (range 0–4); higher scores indicated perceptions of similar or greater harm. Data on menthol smokers’ quitting behaviors were analyzed to identify associations between sum scores and quitting behavior. Data were analyzed using Wilcoxon Rank Sum tests and Spearman Rank Correlation tests. Additional analyses examined whether gender, age, race/ethnicity, education or income moderated the association between sum scores and past 12-month quit attempts.

Menthol smokers were less likely to answer the harm perception questions correctly than nonmenthol smokers. Among menthol smokers, perceived harm of menthol cigarettes was positively associated with past 12–month quit attempts (p = 0.006), use of counseling/behavioral support (p = 0.012), and number of quit attempts (p = 0.004). No demographic characteristics moderated the association between sum scores and past 12-month quit attempts.

Findings suggest that efforts to increase menthol smokers’ perceptions of menthol cigarettes’ harm may potentially increase quitting behaviors. Understanding this association can inform interventions to increase quit attempts.

## Highlights

• There is a need to understand what influences quitting among menthol smokers.
• Links between menthol smokers’ perceived harms of menthol and quitting are unknown.
• Higher menthol harm perceptions were associated with menthol smokers’ quit attempts.
• Findings may inform public health interventions to increase quit attempts.

## 1 Introduction

While studies have documented declining rates of current smoking in Minnesota from 16.1% (±1.2%) in 2010 to 13.8% (±1.2%) in 2018, the proportion of smokers reporting that they currently smoke menthol cigarettes increased from 22.0% (±3.6) to 27.5% (±4.3%) (ClearWay Minnesota and Minnesota Department of Health, 2018). Concerns about this upward trend are particularly germane given the preponderance of evidence demonstrating menthol’s role in easing smoking initiation and reducing quitting success despite more quit attempts (Villanti et al., 2017). Understanding factors that may influence quitting behaviors, such as perceptions of risk associated with menthol smoking, is a public health priority. Neither smokers nor the general public fully understand smoking’s relative risks and, in fact, tend to underestimate these risks (Weinstein et al., Feb 2005, Krosnick et al., 2017). Research demonstrates that if smokers perceive greater health risks due to their smoking, they are more likely to attempt to quit (Costello et al., 2012). However, these studies have focused on smokers’ perceived risks pertaining to specific tobacco-related illnesses (e.g., lung cancer, heart attack), and have not examined this association by menthol smoking status.

Data are mixed on whether menthol smokers believe that menthol cigarettes are more or less harmful than other cigarettes (Glasser et al., 2020, Cohn et al., 2019, Unger et al., 2010, Richter et al., 2008, Richter et al., 2006, Wackowski et al., 2010, Kingsbury et al., May 2020). The tobacco industry’s marketing of the health and medical benefits of menthols led to many consumers believing that menthol cigarettes were less harmful than nonmenthols (Unger et al., 2010, Richter et al., 2008, Wackowski et al., 2010, Kingsbury et al., May 2020, Anderson, 2011). However, other studies have indicated that menthol smokers in fact believe menthol cigarettes are more harmful than nonmenthols (Cohn et al., 2019, Wackowski et al., 2018). Moreover, demographic factors such as age, gender and race may influence harm perceptions (Cohn et al., 2019, Allen et al., 2010). Researchers have identified a need for studies examining associations between perceived harm of menthol cigarettes and quitting behaviors, such as making a quit attempt or using evidence-based cessation treatment (Cohn et al., 2019, Gundersen et al., 2009). To help fill this gap in the evidence, this study assessed whether perceived harms of menthol cigarettes compared to nonmenthol cigarettes was associated with menthol smokers’ quitting behaviors.

## 2 Methods

This study analyzed data from the 2018 Minnesota Adult Tobacco Survey (MATS; N = 6055). MATS is a cross-sectional, random-digit dial survey of Minnesota adults age 18 years and older. The sample was drawn from both landline and cell phone sampling frames. A stratified sampling approach was used to increase inclusion of non-white and Hispanic adults. MATS included questions about smoking status, types of tobacco used (including e-cigarettes), quitting behaviors, secondhand smoke exposure, harm perceptions, and demographics. MATS data were weighted so estimates represent Minnesota’s adult population. A detailed description of the MATS methodology has been published previously (ClearWay Minnesota and Minnesota Department of Health, 2018).

Respondents were categorized as either current menthol smokers (n = 200), current nonmenthol smokers (n = 527), or nonsmokers (n = 5,324). Smoking status definitions aligned with those used by the Centers for Disease Control and Prevention (i.e., current smokers have smoked at least 100 cigarettes in his or her lifetime and now smoke every day or some days; former smokers have smoked at least 100 cigarettes in his or her lifetime but now does not smoke at all; never smokers have not smoked at least 100 cigarettes in his or her lifetime) (National Center for Health Statistics, 2020)

All respondents were asked four questions to assess their perceptions of menthol cigarettes’ harm compared to nonmenthols.

*“Do you agree or disagree with the following statements:*

*Menthols are better for a sore throat than non-menthols*

*Menthols are healthier than non-menthols*

*Menthols contain fewer chemical additives than non-menthols*

*Menthols are less harmful to smokers than non-menthols”*

The questions were informed by the work of Allen et al. (2010). Allen’s scales were focused on African American smokers and included factors about medicinal effects, image, less harmful, tradition, and taste/sensation. Items from the medicinal effects and less harmful scales were used in this study. Since MATS is a general population survey, the above questions were selected for ease of interpretation by smokers and nonsmokers. Questions underwent cognitive testing among current menthol smokers, current nonmenthol smokers, and nonsmokers prior to being fielded. Those participating in the cognitive tests were interviewed individually by telephone. The question was read for the participant to answer. Probes assessed item interpretation and ease of response. All respondents indicated that the intent of the questions was clear and the questions were easy to respond to.

Response options were *agree, disagree, don’t know,* and *refused.* The correct answer for each question was *disagree.* A sum score was created from responses to the four questions (range 0–4), with *disagree* coded as 1, and *agree* and *don’t know* coded as 0. Higher scores indicated the respondent perceived the harms of menthol cigarettes to be similar or greater than nonmenthols.

All current smokers were asked about quitting-related behaviors (any past-12 month quit attempt, number of quit attempts, use of counseling/behavioral support in a quit attempt, use of cessation medications in a quit attempt, use of e-cigarettes in a quit attempt). These data were analyzed to determine whether there was an association between sum scores and quitting behavior. Given that harm perceptions of menthol cigarettes might not be salient for nonmenthol smokers and thus not influence their quitting behavior, these data were analyzed for current menthol smokers only.

Two current menthol smokers did not answer one or more of the harm perception questions and were excluded from the analysis. The final analytic sample consisted of 198 current menthol smokers.

### 2.1 Statistical analysis

As menthol harm perception items and sum scores were not normally distributed, non-parametric methods were used for analysis (Wilcoxon Rank Sum test for dichotomous variables, Spearman Rank Correlation test for continuous variables). Cronbach’s alpha measure of internal consistency was used to calculate the sum score’s reliability (α = 0.77). Due to the analytic sample (n = 198) being a small subset of the larger 2018 MATS sample, analyses were unweighted.

Additional analyses examined whether gender (male/female), age (18–24 vs. 25 +), race/ethnicity (White, Black, Other), education (high school/GED or less vs. more than high school), and income (≤ $35,000, $35,000–$75,000, ≥$75,001) moderated the association between the sum scores and past 12-month quit attempts. An insufficient number of current menthol smokers reported using counseling, medications, or e-cigarettes in a quit attempt to yield reliable results in a moderator analysis. Although there was a positive correlation between the harm perception score and number of quit attempts, the strength of the correlation was weak (rho = 0.21), which likely indicates that there are other factors with a stronger association to number of quit attempts than the harm perception score. Moderators were chosen based on previous studies that examined factors associated with harm perceptions of menthols (Cohn et al., 2019, Unger et al., 2010, Allen et al., 2010). For each potential moderator, data were stratified by subcategory and the association between menthol harm perception score and 12-month quit attempts was compared across subgroups (e.g., male/female) using medians and interquartile ranges. Dissimilarities across subgroups were assessed by checking both the direction of the association and whether there was a meaningful change in the magnitude of the association, taking into consideration the precision of the estimate with smaller sample sizes.

All analyses were conducted using SAS 9.1 (Cary, NC).

The Minnesota Department of Health Institutional Review Board reviewed and approved the study.

## 3 Results

Table 1 reports demographic characteristics. Current menthol smokers were more likely to be Black or report Other race, female, younger, have a high school education/GED or less, and have an annual income of less than $75,000 than either current nonmenthol smokers or nonsmokers.

**Table 1:** Respondent demographics and responses to menthol harm perception questions by smoking status.

	Current menthol smoker		Current nonmenthol smoker		Nonsmoker	
	N = 200		N = 527		N = 5324	
	N	%	N	%	N	%
**Race**						
White	122	62.24%	438	83.75%	4437	84.42%
Black	37	18.88%	11	2.10%	210	4.00%
Other race	37	18.88%	74	14.15%	609	11.59%
**Gender**						
Male	91	45.50%	289	54.84%	2403	45.14%
Female	109	54.50%	238	45.16%	2921	54.86%
**Age**						
18–24 years old	26	13.00%	49	9.30%	362	6.80%
25 years or older	174	87.00%	478	90.70%	4962	93.20%
**Education**						
High school or less	89	44.72%	209	39.81%	1196	22.61%
More than high school	110	55.28%	316	60.19%	4094	77.39%
**Income**						
$35,000 or less	74	42.53%	187	40.48%	1008	21.72%
$35,001 - $75,000	58	33.33%	136	29.44%	1405	30.27%
$75,001 or more	42	24.14%	139	30.09%	2228	48.01%
**Menthols are better for sore throat**^a^						
Don't know	25	12.50%	90	17.14%	1515	28.51%
Agree	42	21.00%	49	9.33%	225	4.23%
Disagree	133	66.50%	386	73.52%	3574	67.26%
**Menthols are healthier**^a^						
Don't know	19	9.60%	48	9.14%	1067	20.07%
Agree	17	8.59%	4	0.76%	104	1.96%
Disagree	162	81.82%	473	90.10%	4145	77.97%
**Menthols contain fewer chemical additives**^a^						
Don't know	40	20.10%	94	17.87%	1659	31.21%
Agree	18	9.05%	22	4.18%	214	4.03%
Disagree	141	70.85%	410	77.95%	3443	64.77%
**Menthols are less harmful**^a^						
Don't know	27	13.57%	49	9.32%	1131	21.28%
Agree	16	8.04%	14	2.66%	148	2.79%
Disagree	156	78.39%	463	88.02%	4035	75.93%
**Sum score menthol harm perception**						
0	18	9.09%	33	6.29%	843	15.88%
1	15	7.58%	20	3.81%	311	5.86%
2	21	10.61%	37	7.05%	420	7.91%
3	42	21.21%	104	19.81%	903	17.02%
4	102	51.52%	331	63.05%	2830	53.33%

Minnesota Adult Tobacco Survey, 2018.

Unweighted N’s and % reported in table.

a Disagree is the correct answer. Refused and Missing are excluded.

Responses to the four harm perception questions provided by current menthol smokers, current nonmenthol smokers, and nonsmokers are reported in Table 1. The distribution of the sum scores is also reported. Current menthol smokers were less likely to answer the questions correctly (i.e., disagree) than current nonmenthol smokers. Nonsmokers were less likely to answer these questions correctly than current menthol smokers, except for the item “menthols are better for a sore throat than nonmenthols.” Nonsmokers were also more likely to respond *don’t know* to each item than either group of current smokers.

Among menthol smokers, perceived harm of menthol cigarettes was positively associated with past 12–month quit attempts (p = 0.006), use of counseling/behavioral support (p = 0.012), and number of quit attempts (p = 0.004) (Table 2). No demographic characteristics moderated the association between harm perceptions and past 12-month quit attempts. In the stratified moderator analysis, none of the demographic characteristics showed a difference in direction of association across subcategory. Medians for all subcategories of the characteristics ranged from 3 to 4 with interquartile range widths from 0 to 3.

**Table 2:** Menthol harm perception score by quitting behavior, among menthol smokers.

Quitting behavior	N	Median	IQR^a^	Wilcoxon z-score	Wilcoxon p-value
*Quit attempts past 12 months: one or more*				−2.74	0.006
No	98	3	2		
Yes	99	4	1		
Missing	1				
*Used stop-smoking class, phone/web/in-person counseling, health professional when quit or during last quit attempt*				2.50	0.012
No	75	4	1		
Yes	14	4	0		
Missing	109				
*Used medications (prescription or nicotine replacement therapy) when quit or during last quit attempt*				−0.61	0.270
No	51	4	1		
Yes	38	4	1		
Missing	109				
*Used e-cigarettes when quit or during last quit attempt*				−1.63	0.051
No	41	4	1		
Yes	20	3.5	2		
Missing	137				
	**N**			**Spearman rho**	**Spearman p-value**
*Number of quit attempts*	193			0.21	0.004
*Missing*	5				

Minnesota Adult Tobacco Survey, 2018

a Interquartile Range

## 4 Discussion

In this observational study, a positive association was observed between menthol smokers who perceived that menthol cigarettes’ harms were similar to or greater than the harms of nonmenthols and quitting-related behaviors (i.e., past-year quit attempts, a greater number of quit attempts, use of counseling or another form of behavioral support in order to quit). While the direction of these findings is consistent with research demonstrating a positive association between smokers’ perceived risk of smoking-related illness and quit attempts, (Costello et al., 2012) evaluating the association between menthol smoking and harm perceptions helps inform the evidence on this topic.

Multiple studies suggest that young adults, women, African Americans, and those with less education or lower income smoke menthol at higher rates (Villanti et al., 2017, Villanti et al., 2016, U.S. Food and Drug Administration, 2011). Our results found no moderation of the association between menthol smokers’ harm perceptions sum scores and our outcome variable of any past-year quit attempts by age, gender, race/ethnicity, education level, and income level. However, this study may have been underpowered to detect small effects due to the relatively small sample size of menthol smokers. More robust studies are needed to more fully investigate potential moderators.

Understanding the association between perceptions of menthol’s harm and quitting behaviors is important to inform both public health interventions and policy. For decades, the tobacco industry marketed menthol as a less harmful alternative (Anderson, 2011, Sutton and Robinson, 2004). Although this study did not assess marketing exposure, a third of smokers responded incorrectly that menthol cigarettes were better for a sore throat and over 20% responded incorrectly that menthol cigarettes are less harmful. Robust counter marketing campaigns offer an option to increase awareness of menthol’s harms among menthol smokers. Increased awareness may then lead to greater interest in and attempts to quit. A collateral benefit of such campaigns would be the opportunity to increase awareness of menthol’s harms among the general public. In this study, nonsmokers were much more likely to respond “don’t know” to the harm perception questions than smokers. Elevating public awareness has the potential to contribute to changing social norms about the harms of menthol and spur greater support of policies restricting or banning the sale of menthol tobacco products.

## 5 Study limitations

Since the data analyzed are from a cross-sectional study, the observed associations between harm perception scores and quitting-related behaviors are not causal. The study included four harm perception questions that were informed by the literature. An area for further study is validating existing harm perception scales among the general population versus solely among smokers and menthol smokers, as understanding similarities and differences between groups may inform efforts to increase public awareness about menthol. Small sample sizes reduced our ability to conduct additional subgroup analyses (e.g., moderator analyses of use of counseling, levels of education and income). Further research is needed on other menthol tobacco products, including e-cigarettes, as well as follow-up studies to assess quit outcomes. Additionally, findings from this study may be unique to Minnesota. Despite these limitations, the results offer preliminary insights on the association between perceived harm of menthol cigarettes on quitting behavior among menthol smokers.

## 6 Conclusions

Study findings suggest that efforts to increase menthol smokers’ perceived harm of menthol cigarettes may have the potential to increase quitting behaviors. Further studies are warranted to test whether increasing menthol smokers’ perceived harm of menthol cigarettes will in fact increase quitting behaviors, and to identify whether particular harm perceptions are the most salient and result in the greatest behavior change by menthol smokers.

## Author statement

All authors contributed to the study design. Professional Data Analysts, Inc. collected and analyzed the data, and led the interpretation of findings. All authors contributed to the writing of this report and decided to submit the article for publication.

## CRediT authorship contribution statement

**Paula A. Keller:** Conceptualization, Methodology, Writing - original draft, Writing - review & editing, Supervision, Project administration. **Joanne D’Silva:** Conceptualization, Methodology, Writing - original draft, Writing - review & editing, Supervision. **Rebecca K. Lien:** Methodology, Formal analysis, Writing - original draft, Writing - review & editing. **Raymond G. Boyle:** Conceptualization, Methodology, Writing - review & editing. **John Kingsbury:** Conceptualization, Writing - review & editing. **Erin O’Gara:** Conceptualization, Writing - review & editing.

## Conflicts of interest

R. Lien is employed by Professional Data Analysts, an independent consulting firm contracted by ClearWay Minnesota for statistical consulting, including this study. R. Boyle is an independent consultant contracted by ClearWay Minnesota for analysis and writing, including this study. P. Keller, J. D’Silva, E. O’Gara, and J. Kingsbury have no financial disclosures.

## References

[b0005] Allen B., Cruz T.B., Leonard E., Unger J.B. (2010). Development and validation of a scale to assess attitudes and beliefs about menthol cigarettes among African American smokers. Eval. Health Prof..

[b0010] Anderson S.J. (2011). Marketing of menthol cigarettes and consumer perceptions: a review of tobacco industry documents. Tob. Control.

[b0015] ClearWay Minnesota and Minnesota Department of Health. Minnesota Adult Tobacco Survey: Tobacco Use in Minnesota: 2018 Update. January 2019. http://clearwaymn.org/mats/reports/.

[b0020] Cohn A.M., Rose S.W., Ilakkuvan V. (2019). Harm perceptions of menthol and nonmenthol cigarettes differ by brand, race/ethnicity, and gender in US adult smokers: results from PATH Wave 1. Nicotine Tob. Res..

[b0025] Costello M.J., Logel C., Fong G.T., Zanna M.P., McDonald P.W. (2012). Perceived risk and quitting behaviors: results from the ITC 4-country survey. Am. J. Health Behav..

[b0030] Glasser A.M., Barton A., Rath J., Simard B., Rose S.W., Hair E., Vallone D. (2020). Perceptions of use patterns and health consequences associated with mentholated cigarettes among U.S. adults. Health Educ. Behav..

[b0035] Gundersen D.A., Delnevo C.D., Wackowski O. (2009). Exploring the relationship between race/ethnicity, menthol smoking, and cessation, in a nationally representative sample of adults. Prev. Med..

[b0040] Kingsbury J.H., Mehrotra K., D'Silva J., Nichols E., Tripp R., Johnson D. (May 2020). Perceptions of menthol cigarettes and reasons for unsuccessful quits in an African American community sample. J. Immigr. Minor Health..

[b0045] Krosnick J.A., Malhotra N., Mo C.H. (2017). Perceptions of health risks of cigarette smoking: a new measure reveals widespread misunderstanding. PLoS One.

[b0050] National Center for Health Statistics. National Health Interview Survey, Glossary, General Concepts. Accessed October 15, 2020, https://www.cdc.gov/nchs/nhis/tobacco/tobacco_glossary.htm.

[b0055] Richter P., Beistle D., Pederson L., O'Hegarty M. (2008). Small-group discussions on menthol cigarettes: listening to adult African American smokers in Atlanta, Georgia. Ethnicity Health.

[b0060] Richter P.A., Pederson L.L., O'Hegarty M.M. (2006 May-Jun 2006,). Young adult smoker risk perceptions of traditional cigarettes and nontraditional tobacco products. Am. J. Health Behav..

[b0065] Sutton C.D., Robinson R.G. (2004). The marketing of menthol cigarettes in the United States: populations, messages, and channels. Nicotine Tob. Res.

[b0070] U.S. Food and Drug Administration Tobacco Products Scientifıc Advisory Committee. Report and recommendations on the impact of the use of menthol in cigarettes on the public health. March 23, 2011. Accessed October 15, 2020. www.fda.gov/downloads/AdvisoryCommittees/CommitteesMeetingMaterials/TobaccoProductsScientifıcAdvisoryCommittee/UCM247689.pdf.

[b0075] Unger J.B., Allen B., Leonard E., Wenten M., Cruz T.B. (2010). Menthol and non-menthol cigarette use among Black smokers in Southern California. Nicotine Tob. Res..

[b0080] Villanti A.C., Mowery P.D., Delnevo C.D., Niaura R.S., Abrams D.B., Giovino G.A. (2016). Changes in the prevalence and correlates of menthol cigarette use in the USA, 2004–2014. Tob. Control.

[b0085] Villanti A.C., Collins L.K., Niaura R.S., Gagosian S.Y., Abrams D.B. (2017). Menthol cigarettes and the public health standard: a systematic review. BMC Public Health.

[b0090] Wackowski O.A., Delnevo C.D., Lewis M.J. (2010). Risk perceptions of menthol cigarettes compared with nonmenthol cigarettes among New Jersey adults. Nicotine Tob. Res..

[b0095] Wackowski O.A., Evans K.R., Harrell M.B. (2018). In their own words: Young adults' menthol cigarette initiation, perceptions, experiences and regulation perspectives. Nicotine Tob Res..

[b0100] Weinstein N.D., Marcus S.E., Moser R.P. (Feb 2005). Smokers' unrealistic optimism about their risk. Tob. Control.

